# The impact of *Caralluma munbyana* extracts on *Streptococcus mutans* biofilm formation

**DOI:** 10.3389/fdmed.2025.1575161

**Published:** 2025-06-04

**Authors:** Turki Alshehri, Israa Alkhalifah, Areeb Alotaibi, Alaa F. Alsulaiman, Abdullah Al Madani, Basil Almutairi, Abdulrahman A. Balhaddad

**Affiliations:** ^1^ Department of Substitutive Dental Sciences, College of Dentistry, Imam Abdulrahman Bin Faisal University, Dammam, Saudi Arabia; ^2^ College of Dentistry, Imam Abdulrahman Bin Faisal University, Dammam, Saudi Arabia; ^3^ Department of Dentistry, King Fahad Military Medical Complex, Dhahran, Saudi Arabia; ^4^ Dental Hospital, College of Dentistry, Imam Abdulrahman Bin Faisal University, Dammam, Saudi Arabia; ^5^ Department of Restorative Dentistry, Division of Operative Dentistry, King Saud University, Riyadh, Saudi Arabia; ^6^ Department of Restorative Dental Sciences, College of Dentistry, Imam Abdulrahman Bin Faisal University, Dammam, Saudi Arabia

**Keywords:** absorbance, biofilms, *caralluma munbyana*, caries, *streptococcus mutans*

## Abstract

**Background/objectives:**

*Caralluma* plants have a wide range of anti-inflammatory and antimicrobial activities. This study aims to assess the antibacterial effect of water, methanol, and ethanol extracts of *Caralluma munbyana* against *Streptococcus mutans* biofilms.

**Methods:**

Three extracts of *C. munbyana* were prepared using water, methanol, and ethanol. Multiple concentrations ranging between 2.93 and 93.75 mg/ml were achieved, alongside a control group with no extract, and incubated with an overnight culture of *S. mutans*. In the following day, the total absorbance was measured at 595 nm. Then, the biofilms were fixed and stained with 0.5% crystal violet to measure the biofilm absorbance at 490 nm. One-way ANOVA and Tukey's *post-hoc* tests were applied to identify which specific concentrations differed from the control.

**Results:**

*C. munbyana* methanol and ethanol extracts significantly affected the total absorbance of *S. mutans* (*P* ≤ 0.001) at 46.87 and 93.75 mg/ml. For biofilm inhibition, *C. munbyana* water extract was effective (*P* ≤ 0.001) in reducing the biofilm growth at 23.44 (1.34 ± 0.08), 46.87 (1.31 ± 0.15), and 93.75 (1.04 ± 0.07) mg/ml when compared to the control (1.58 ± 0.11). More reduction was observed among methanol and ethanol extracts, as *C. munbyana* methanol extract significantly (*P* ≤ 0.001) inhibited the *S. mutans* biofilm growth at 23.44 (0.99 ± 0.15), 46.87 (0.12 ± 0.02), and 93.75 (0.09 ± 0.01) mg/ml. Similarly, *C. munbyana* ethanol extract's biofilm inhibition was observed at the concentrations of 23.44 (0.45 ± 0.12), 46.87 (0.10 ± 0.02), and 93.75 (0.09 ± 0.04) mg/ml.

**Conclusion:**

These findings suggest that *C. munbyana* possesses antibacterial properties against *S. mutans* biofilms, particularly through its methanol and ethanol extracts.

## 1 Introduction

Dental caries stands out as one of the most prevalent oral diseases worldwide (1). It is defined as a chronic infectious disease caused by cariogenic bacteria that utilize available carbohydrates to produce acids, leading to the destruction of tooth structure (2). This multifactorial disease is affected by oral hygiene practice, consumed diet, and the cariogenicity of the oral microbes (3). Dental caries pathogenesis involves a complex interaction between several microbes with *Streptococcus mutans* (*S. mutans*) among the main key pathogens (3). The cariogenicity of *S. mutans* is related to many virulence factors, including the ability of this bacterium to adhere to the tooth structure, form a biofilm with other caries-related pathogens, produce lactic acid, and survive in a highly acidic environment (4).

*S. mutans* utilizes multiple necessary enzymes to cause dental caries. Among different enzymes, glycosyltransferase plays a critical role by enabling glucans synthesis from dietary sugars, which allows *S. mutans* to attach to the tooth surfaces and form biofilms (5). Fructosyltransferase is another enzyme that helps produce fructans, which aid as a reserve of energy for the microorganisms, supporting their survival and growth (6). Furthermore, *S. mutans* exert acidogenic enzymes to ferment carbohydrates and produce acid to demineralize the tooth structure (7, 8). Without proper intervention and the increased acidity at the biofilm-tooth interface, demineralization will occur due to the loss of calcium and phosphate minerals from tooth structure, which can subsequently lead to tooth cavitation (9).

Nowadays, several mechanical and chemical methods are available to control plaque accumulation and biofilm development, such as dentifrices, dental floss, and mouthwashes (10, 11). While using these oral hygiene products is critical and must not be neglected, there is a need to design other adjunctive approaches to control biofilm-triggered oral diseases. Throughout the ages of humanity, the utilization of herbal products as a form of medicine has been an integral part of human history (12). Natural resources of medicinal plants are rich in biological components that include antibacterial properties (13). Besides, the use of herbal products as medicine has potential implications for addressing the issue of bacterial resistance (14, 15), which is a growing concern in modern healthcare.

Several herbal and natural products have been extensively studied in recent years to control oral diseases (16), and a wide range of plants have demonstrated antimicrobial activity (17). For example, *Carum copticum* (18), *Salvadora persica*, and other herbs such as cloves, garlic, and liquorice (19) have been found effective against *S. mutans*. In addition, green tea, Aloe vera, sesame, Triphala and many other plant-derived compounds were found effective to control plaque accumulation and prevent the onset of gingivitis and aphthous stomatitis (20). Therefore, natural products can serve as alternative treatments to help prevent the formation of dental caries. One of the most recognized plants that demonstrated effective and promising results is *Caralluma* (21). *Caralluma* is a species of plant that belongs to the *Asclepiadaceae* family and is found in various regions, including Africa, Saudi Arabia, and India (21). Multiple studies addressed the pharmacological prominence of *Caralluma* in different medicinal applications, which include diabetes, cancer, muscle pain, and inflammation (22, 23). Besides, *Caralluma* has demonstrated significant anti-inflammatory action via the reduction of oxidative stress and the inhibition of pro-inflammatory cytokines and related mediators (24, 25).

*Caralluma* species have been also used as antimicrobial agents against different types of pathogens, mainly because of the presence of pregnane glycosides, stigmasterol, flavonoids, and other further constituents (26–28). In one study, 0.625, 0.313, and 0.156 mg/ml of *Caralluma quadrangula* extracts were found effective in inhibiting the biofilm growth of methicillin-resistant *Staphylococcus aureus* and multidrug-resistant *Acinetobacter baumannii, in vitro* and *in vivo,* using an animal model (29). Another study demonstrated the capability of *Caralluma lasiantha* extracts to reduce the growth of *Staphylococcus aureus*, *Escherichia coli*, *Streptococcus* Sp., *Bacillus subtilis*, *Enterobacter aerogenes*, and *Klebsiella pneumoniae* (30). The antifungal properties of *Caralluma* were also demonstrated in one investigation, as the *Candida albicans* growth was inhibited following exposure to different *Caralluma europaea* extracts (31). It has been suggested that the release of oxalic acid and propanoic acid from *C. europaea* disrupt the cell membrane and metabolic activities of *C. albicans* (31).

Given the existing evidence of the therapeutic and antibacterial properties of various *Caralluma* species, this study aims to explore the antibacterial effect of *Caralluma munbyana* for the first time as an alternative or adjunct natural therapeutic agent to control dental caries. We seek to investigate its potential to inhibit the growth of caries-related pathogens as a strategy to prevent dental caries. Specifically, this paper examines the inhibitory effect of *C. munbyana* against *Streptococcus mutans* in both total and biofilm growth, utilizing three different extracts: water, methanol, and ethanol. We hypothesize that the concentration of *C. munbyana* and the type of extract will significantly influence its antibacterial activities.

## 2 Materials and methods

### 2.1 Sample size calculation and study design

Prior studies (32–34) indicate that the standard deviation for absorbance measurements related to biofilm formation is approximately 0.15. Consequently, this study was designed to achieve 80% power to identify a significant difference at a 5% significance level. This involved conducting three repeated experiments with 3–4 samples each, leading to a total of 9–12 samples per group.

*C. munbyana* plants were collected from a local store in the Southern area of Saudi Arabia (Al Dunya Gardens Agricultural Inc., Al Namas, Saudi Arabia). Fifteen grams of the plant were grinded and placed in three different tubes containing distilled water, pure methanol, or pure ethanol, resulting in a final concentration of 375 mg/ml. This final concentration was the maximum to be achieved without having the plant floating from the selected extracts. The tubes were incubated for one week in 4°C refrigerator to allow the extraction of the chemical components of *C. munbyana*. Then, the distilled water, methanol, and ethanol extract of *C. munbyana* was diluted with brain-heart infusion (BHI) broth supplemented with 2 wt.% of sucrose to achieve different concentrations of 93.75, 46.875, 23.44, 11.72, 5.85, and 2.93 mg/ml. The original extracts were diluted to eliminate the possibility of antibacterial action caused by ethanol and methanol themselves. In addition, the selected concentrations were investigated to explore a wide range of concentrations, which was achieved in similar previous studies (32, 35, 36).

### 2.2 Effect of C. munbyana extracts on S. mutans growth

**Figure 1:**
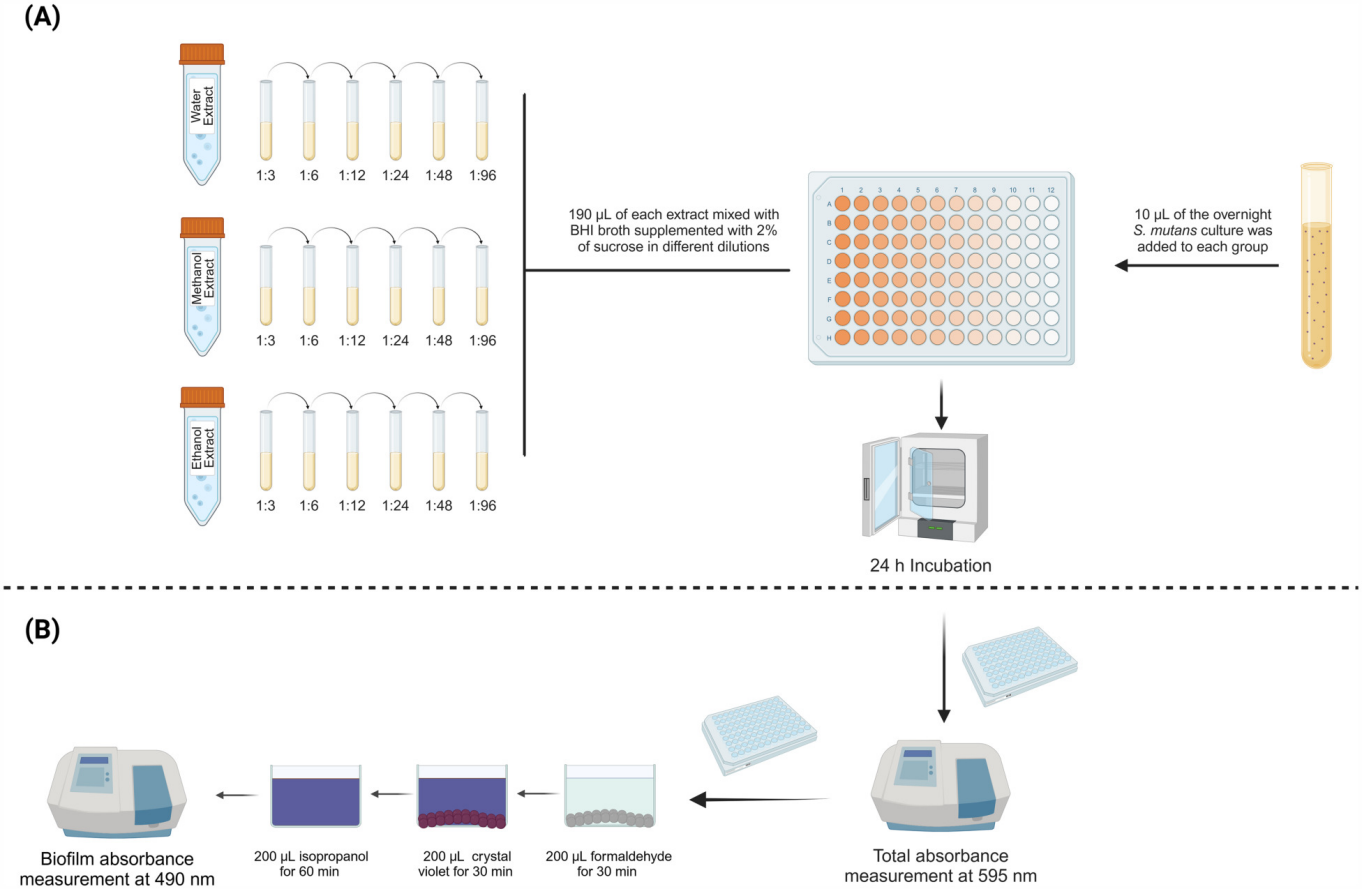
Schematic overview of study design. **(A)** S. mutans were grown overnight in 5 ml of brain-heart infusion (BHI) broth and then incubated with different concentrations of water, methanol, and ethanol extracts of *C. munbyana* for another 24 h. **(B)** On the following day, the total and biofilm absorbance were measured at 595 and 490 nm, respectively.

The methodology for this study is illustrated in Figure 1. *S. mutans* UA159 (ATCC 700610, American Type Culture Collection, Rockville, MD, USA) was grown in 5 ml of Brain Heart Infusion (BHI) and incubated overnight for 24 h. BHI is a nutrient-rich liquid medium that is commonly used for the growth of pathogens including bacteria and fungi. The main two components of BHI are brain extract and heart infusion, which provide essential growth factors and proteins. On the subsequent day, 190 µl of each concentration from each extract was added to the wells of a sterile 96-well flat-bottom microtiter plate. Then, 10 µl of the overnight *S. mutans* culture, approximately 10^^^6 colony-forming units (CFU)/ml, was introduced into each well. The plates were incubated for another 24 h. The total absorbance of the culture, including both planktonic cells and biofilms, was measured at 590 nm using a spectrophotometer (SpectraMax M5, Molecular Devices, Sunnyvale, CA, USA) (32–34). Afterward, the planktonic cells were discarded, leaving only the attached biofilm. To fix the biofilm cells, 200 µl of 10% formaldehyde was added to each well and incubated for 30 min. The biofilms were rinsed three times with deionized water. Next, 200 µl of 0.5% crystal violet dye was added to stain the biofilm, followed by another three rinses with deionized water. To extract the crystal violet, 200 µl of 2-isopropanol was added and incubated for one hour. The biofilm formation was then quantified using the spectrophotometer at 490 nm (32–34). Two control groups were included in the study: negative control with only the overnight culture of *S. mutans* in BHI supplemented with 2% sucrose, and a sterility control group containing only BHI growth media to confirm the absence of microbial contamination. The minimum inhibitory concentration (MIC) and the minimum biofilm inhibitory concentration (MBIC) values were determined as the lowest concentration capable of inhibiting visible bacterial growth and biofilm growth, respectively, after incubation at 37°C for 24 h (37, 38).

### 2.3 Statistical analysis

Data (means ± standard deviations) were presented as descriptive data representing a minimum of 3 biological replicates. Shapiro–Wilk test was used to examine the data normality. One-way ANOVA and Tukey tests were applied to compare the effects of *C. munbyana* extracts on *S. mutans* biofilm and total growth (Sigma Plot 12.0; SYSTAT). A *p*-value <0.05 was considered statistically significant.

## 3 Results

**Figure 2:**
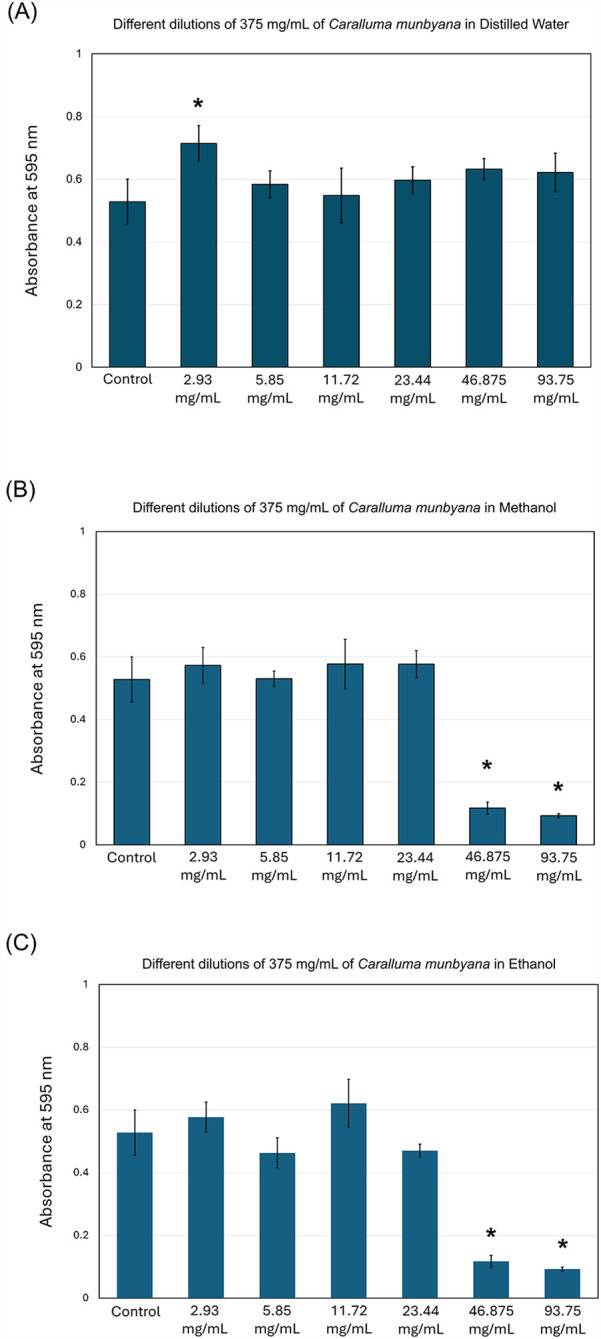
Effect of the *Caralluma munbyana* **(A)** water **(B)** methanol, and **(C)** ethanol extracts on *Streptococcus mutans* total growth. Each group consisted of 3 wells, and the experiment was repeated three times (*n* = 9). Asterisks indicate a significant difference compared to the control samples with no treatment.

**Figure 3:**
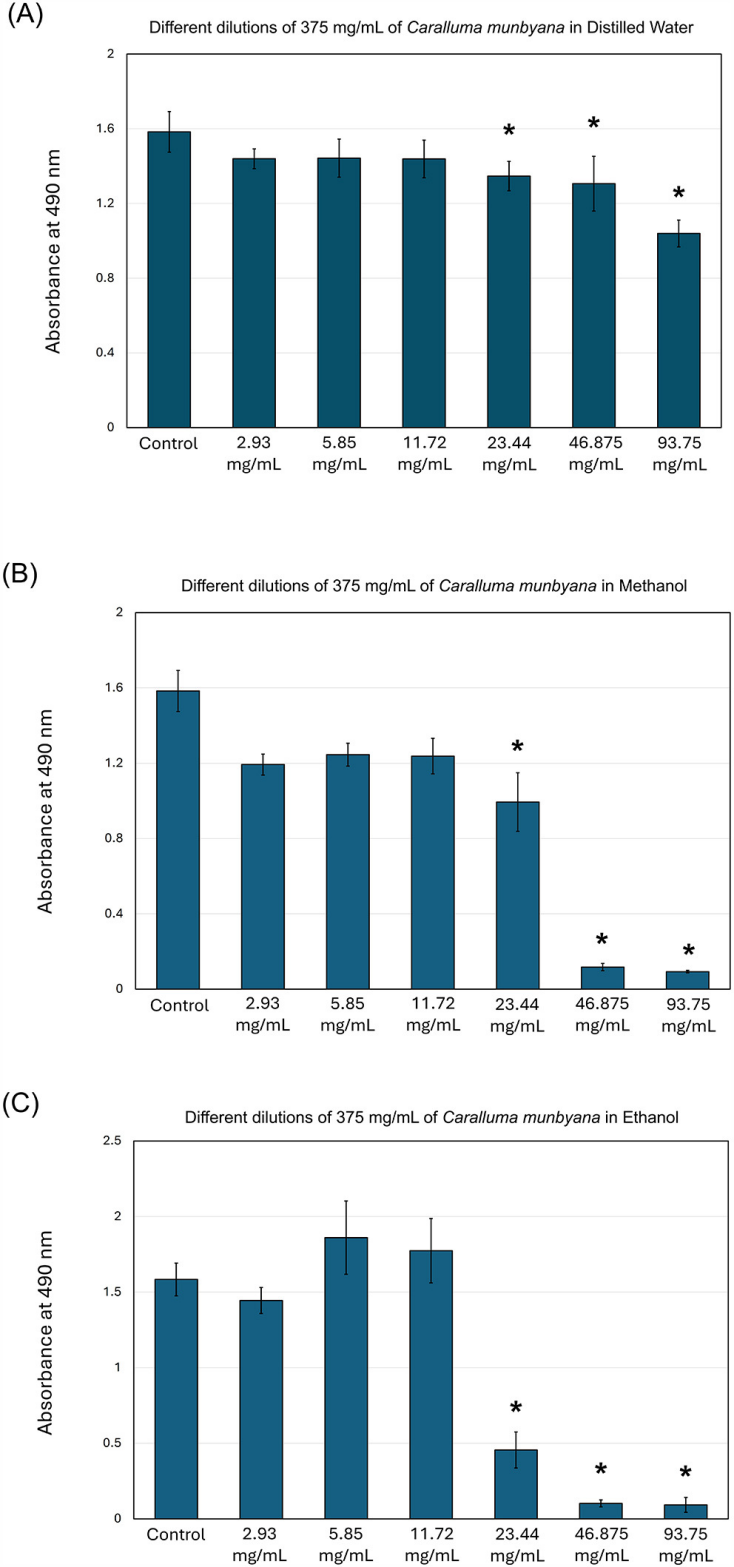
Effect of the *Caralluma munbyana* **(A)** water **(B)** methanol, and **(C)** ethanol extracts on *Streptococcus mutans* biofilm formation. Each group consisted of 3 wells, and the experiment was repeated three times (*n* = 9). Asterisks indicate a significant difference compared to the control samples with no treatment.

The results of this study indicate that *Caralluma munbyana* significantly affected the total absorbance of *S. mutans* in the methanol and ethanol extracts (*P* ≤ 0.05) at the concentrations of 23.44, 46.87, and 93.75 mg/ml (Figure 2). No effect was observed in total absorbance following the exposure to different concentrations of water extract, except a significant increase at the 2.93 mg/ml concentration. The minimum inhibitory concentrations (MIC) of the methanol and ethanol extracts was 46.875 mg/ml. In Figure 3, the antibiofilm effects of *Caralluma munbyana* can be observed in the three extracts. In Figure 3A, *Caralluma munbyana* water extract was found to be significantly effective (*P* ≤ 0.001) in reducing the biofilm growth at 23.44 (1.34 ± 0.08), 46.87 (1.31 ± 0.15), and 93.75 (1.04 ± 0.07) mg/ml when compared to the control (1.58 ± 0.11).

When *Caralluma munbyana* methanol and ethanol extracts were assessed, the 23.44, 46.87, and 93.75 mg/ml significantly (*P* ≤ 0.001) reduced the biofilm growth. In Figure 3B, *Caralluma munbyana* methanol extract significantly (*P* ≤ 0.001) inhibited the *S. mutans* biofilm growth at 23.44 (0.99 ± 0.15), 46.87 (0.12 ± 0.02), and 93.75 (0.09 ± 0.01) mg/ml. Finally, when the *Caralluma munbyana* ethanol extract was assessed (Figure 3C), the biofilm inhibition was observed at the concentration of 23.44 (0.45 ± 0.12), 46.87 (0.10 ± 0.02), and 93.75 (0.09 ± 0.04) mg/ml, while the other concentrations were comparable to the control. The minimum biofilm inhibitory concentrations (MBIC) for the water extract was 93.75 mg/ml and 46.875 mg/ml for the methanol and ethanol extracts.

## 4 Discussion

Herbal medicines derived from natural sources to enhance the quality of life and achieve various health benefits have significantly increased globally (39). A diverse array of plant-derived preparations, including herbs, are employed for the prevention and treatment of diseases (12, 15). In this study, the hypothesis was accepted as the type of extract and the concentration were determinant factors in modulating the antibacterial activities of *C. munbyana.* Multiple *Caralluma* species have shown a wide spectrum of antibacterial activity against fungi, Gram-positive, and Gram-negative bacteria (29–31, 40, 41). Here, for the first time, we intended to explore the potential antibacterial properties of *C. munbyana*. In particular, we utilized *C. munbyana* to inhibit the growth of caries-related pathogens, *S. mutans*. This biofilm inhibition was achieved at different concentrations using three different extracts. It was found that methanol and ethanol extracts at high concentrations were associated with more bacterial growth inhibition, suggesting that both the extract type and its concentration are critical factors for achieving antimicrobial efficacy against *S. mutans*.

The ability of bacteria to form biofilms on both living and non-living surfaces contributes to chronic infections that can harm hard and soft tissues (42, 43). In dentistry, biofilm-triggered diseases contribute to the onset of two main global chronic diseases, dental caries and periodontal diseases (1, 44). As a result, designing approaches to control and limit the onset of bacterial biofilms is highly needed and presents an alternative strategy for combating bacterial infections to prevent and treat such diseases. Our findings illustrated that the antibacterial properties were improved when using methanol and ethanol extracts, which aligned with previous studies showing that alcoholic extracts are more potent than water extracts (32, 45). This is mainly due to the ability of methanol and ethanol to extract therapeutic chemicals more efficiently compared to distilled water.

Biofilms present a significant challenge in the fight against bacterial infections compared to planktonic bacteria due to their complex structure and behavior (43). Unlike planktonic bacteria, which exist as individual cells in a free-floating state, biofilms consist of communities of bacteria encased in a protective extracellular matrix (43). This matrix not only shields the bacteria from the immune system but also impedes the penetration of antibacterial agents, making treatment more difficult (46, 47). Furthermore, the bacteria within biofilms often exhibit altered metabolic rates, leading to increased resistance to antibiotics that would otherwise be effective against their planktonic counterparts. This resilience is compounded by the presence of diverse bacterial species within a biofilm, which can exchange genetic material, including resistance traits, further complicating treatment efforts (46, 47). Consequently, infections associated with biofilms are often persistent and harder to eradicate, necessitating the development of specialized strategies to target these resilient communities (8).

In this study, we found that the 93.75, 46.875, and 23.44 mg/ml of water, methanol, and ethanol extracts significantly inhibited biofilm growth, with the greatest inhibition observed at the concentrations of 46.875 and 93.75 mg/ml of the methanol and ethanol extracts. Notably, the only concentration that inhibited the biofilm growth of *S. mutans* without compromising total bacterial growth was 23.44 mg/ml of the ethanol extract. This suggests that this specific concentration and extract can prevent biofilm attachment without killing the targeted bacteria. Such an effect could be advantageous, as it allows for the prevention of biofilm development while preserving commensal and planktonic microorganisms, thereby maintaining a balanced microbial community in the oral cavity. However, this does not mean that the other concentrations with bactericidal effects are unimportant; they may still be useful for different clinical applications.

Methanol and ethanol extracts of plants are often more antibacterial than water extracts because many antibacterial compounds, such as phenolics, flavonoids, and alkaloids, are more soluble in organic solvents like methanol and ethanol than in water (32, 48, 49). This higher solubility allows for better extraction of these active compounds. In addition, methanol and ethanol are less polar than water, which enables them to extract a broader range of phytochemicals, including those that might not dissolve well in water (32, 48, 49). This can enhance the overall antibacterial activity of the extract. In this study, methanol and ethanol extracts demonstrated more antibiofilm action against *S. mutans* compared to the water extract, suggesting that alcoholic extracts are more effective in extracting the bioactive compounds of *C. munbyana*.

The superior properties of *Caralluma* primarily arise from the presence of pregnane glycosides, stigmasterol, flavonoids, and other constituents, which enhance its antimicrobial activities and play a crucial role in inhibiting bacterial biofilms (29). The mechanisms of action of these compounds have been studied to a limited extent; however, it is believed that they may interfere with nucleic acid synthesis and disrupt cell membrane function (50, 51). Our findings agree with previous papers showing that *Caralluma* species have antimicrobial properties (26–28). Moreover, *C. quadrangula* extracts at low concentrations significantly inhibited the biofilm growth of methicillin-resistant *S. aureus* and multidrug-resistant *A. baumannii, in vitro* and *in vivo,* using an animal model (29). The antifungal properties of *Caralluma* were also investigated. In one study, *C. europaea* extracts was found effective to inhibit the growth of *C. albicans*, mainly by the release of oxalic acid and propanoic acid (31). Moreover, *Caralluma* species were found effective against oral pathogens. *C. lasiantha* extracts were found effective to reduce the growth of *S. aureus* and *Streptococcus* Sp. (30). In another study, *Caralluma indica* extract significantly inhabited the biofilm growth of *S. aureus* and *C. albicans* (52)*.* It was suggested that bioactive compounds, such as 1-nonadecene, n-hexadecanoic acid, tetradecane, 1-heptadecene, and dibutyl phthalate, can be released and contribute to the antimicrobial activities of *C. indica*.

In this study, the impact of *Caralluma* against caries-related pathogens has been demonstrated for the first time. The results obtained here may imply that the antibiofilm properties of *C. munbyana* may play an important role in preventing dental plaque formation. In terms of clinical practice, daily use of oral hygiene products, such as toothpaste, is essential for maintaining oral health. Multiple manufacturers and multinational companies have included herbal ingredients in their oral hygiene products (53). Therefore, *Caralluma* species and their chemical ingredients could be incorporated into oral dentifrice as a strategy to prevent plaque accumulation and biofilm formation.

In addition, *Caralluma* extracts could be incorporated into mouthwashes as a strategy to prevent the growth of pathogenic bacteria causing caries and periodontal diseases. Such an approach could be an alternative option to limit the use of alcohol-containing mouthwashes, which have been linked to soft tissue irritation and oral cancer (54). *Caralluma* species could be used to reduce soft tissue irritation due to the presence of flavonoids. *Caralluma adscendens* exhibited antifungal activity against *C. albicans* in rat skin, and the prepared cold cream in the same study showed good stability and permeability without any signs of irritation (55). Consequently, customizing oral lubricants and creams from such plants will help reduce oral gingival inflammation and could be used as a potential approach to prevent denture stomatitis among denture wearers. More recently, postbiotics have been introduced as a strategy ton control different oral diseases (56). Integrating different *Caralluma* species with postbiotics could deliver a synergetic and effective approach to control different oral diseases.

Finally, oral health care providers need to exercise caution when interpreting the data from this study, as it has some limitations. First, the study focused solely on one type of *Caralluma*, *C. munbyana*. There are numerous species within the Caralluma genus, each potentially possessing different phytochemical profiles and antibacterial properties. Exploring additional species could yield valuable insights into their respective antibacterial activities and their suitability for oral health applications. Second, this study exclusively examined the antibacterial effects of *C. munbyana* against *Streptococcus mutans*. While *S. mutans* is a significant contributor to dental caries, other pathogens in the oral microbiome also play critical roles in oral health. Future research should investigate the effects of *C. munbyana* against a broader range of dental pathogens, more preferably multi-species biofilms, to provide a more comprehensive understanding of its antibacterial potential. Finally, this study did not account for varying environmental conditions that could affect the antibacterial efficacy of *C. munbyana*. Factors such as pH, temperature, and the presence of other microorganisms can significantly influence the outcomes of antibacterial studies. To validate the results and assess the applicability of *C. munbyana* in dental care, more clinically relevant studies are necessary. Utilizing clinical translation models will help to determine the effectiveness of *C. munbyana* in actual oral environments and its potential for integration into clinical practice. Future studies may employ Gas chromatography-mass spectrometry (GC-MS) and high-performance liquid chromatography (HPLC) to identify the specific compounds released from *C. munbyana* within the investigated extracts. Future research should aim to replicate findings under diverse environmental conditions utilizing clinical translation models to better understand the practical applications of *C. munbyana*. Additionally, conducting toxicity assays in future studies is crucial to assessing the safety of the plant for potential therapeutic use.

## 5 Conclusion

This study demonstrates that *C. munbyana* exhibits significant antibacterial activity against *S. mutans*, particularly through its methanol and ethanol extracts. The results indicate that these extracts effectively reduce the total absorbance of *S. mutans*, with statistically significant differences observed at high concentrations, highlighting their potential as powerful antimicrobial agents. Notably, while the water extract showed no significant impact on total absorbance, it did reduce biofilm growth at certain concentrations, suggesting that it may still play a role in modulating bacterial colonization in oral environments. These findings reinforce the potential of *C. munbyana* as an effective agent in managing oral biofilms, which are critical in the development of dental caries and other oral health issues. The varying efficacy of the different extracts also points to the importance of extraction methods in maximizing the antibacterial properties of natural compounds. Future research should explore the mechanisms behind these effects and investigate the potential for developing *C. munbyana* extracts into practical applications for oral health care.

## Data Availability

The original contributions presented in the study are included in the article/Supplementary Material, further inquiries can be directed to the corresponding author.
